# Plant neighbourhood diversity effects on leaf traits: A meta‐analysis

**DOI:** 10.1111/1365-2435.14441

**Published:** 2023-09-29

**Authors:** Juri A. Felix, Philip C. Stevenson, Julia Koricheva

**Affiliations:** ^1^ Department of Biological Sciences Royal Holloway University of London Egham UK; ^2^ Royal Botanic Gardens Kew UK

**Keywords:** associational effects, BEF, defence, insect herbivore, leaf traits, meta‐analysis neighbourhood diversity

## Abstract

1. Leaf traits often vary with plant neighbourhood composition, which in turn may mediate plant susceptibility to herbivory. However, it is unknown whether there are any common patterns of change in leaf trait expression in response to neighbourhood diversity, and whether these responses confer increased resistance or susceptibility to herbivores.
2. We used meta‐analysis to combine data from 43 studies that examined the influence of neighbourhood diversity on eight physical and chemical leaf traits that could affect herbivory. All leaf traits apart from leaf thickness were highly plastic and exhibited significant differences between plant monocultures and species mixtures, but the direction of effect was variable. Leaf toughness was the only trait that displayed a significant decrease with plant diversity, whereas specific leaf area (SLA) and leaf nitrogen were both marginally increased in species mixtures.
3. The magnitude and direction of leaf trait responses to neighbourhood diversity were independent of plant density and phylogenetic diversity, but changes in SLA correlated positively with plant species richness. SLA was also significantly increased in experimental studies, but not in observational studies, while neighbourhoods containing nitrogen‐fixers were associated with increased leaf nitrogen and reduced phenolics. When studies on the over‐represented species *Betula pendula* were removed from the analysis, the effect of neighbourhood diversity on leaf toughness became nonsignificant, but phenolics were significantly reduced in diverse neighbourhoods composed of mature trees, and marginally reduced in species mixtures across all studies.
4. Increases in plant neighbourhood diversity are often associated with reductions of herbivory, although in some cases, the reverse occurs, and plants growing in species mixtures are found to suffer greater herbivory than those in monocultures. This study offers a potential explanation for the latter phenomenon, as our results show that leaf trait expression is highly plastic in response to neighbourhood diversity, and in certain cases could lead to increased leaf quality, which in turn could promote greater rates of herbivory.

Read the free Plain Language Summary for this article on the Journal blog.

## Graphical abstract

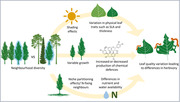

## 1 INTRODUCTION

Plants growing in mixed‐species neighbourhoods are often subject to lower rates of herbivory than those growing in monocultures (Jactel et al., 2021). The mechanisms frequently attributed to this phenomenon include reduced host plant apparency and increased regulation of herbivores by predators and parasitoids (Barbosa et al., 2009; Guyot et al., 2016; Jactel et al., 2021; Letourneau et al., 2011; Root, 1973; Stemmelen et al., 2022). However, these mechanisms are unable to account for the results of numerous studies that have documented increased rather than decreased herbivory in diverse neighbourhoods, which suggests that additional factors are involved in determining the strength and direction of plant neighbourhood effects on herbivores (Barbosa et al., 2009; Berthelot et al., 2021; Jactel et al., 2021; White & Whitham, 2000). One such factor that has been increasingly explored is the intraspecific variation in physical and chemical leaf traits of the focal plant in different neighbourhoods, that can in turn influence leaf quality and rates of herbivory (Mraja et al., 2011; Poeydebat et al., 2020; Rosado‐Sánchez et al., 2018a). Understanding the patterns of leaf trait variation in heterospecific vs conspecific neighbourhoods may offer additional insights into the variability of neighbourhood diversity effects on herbivores, as well as other processes that are mediated by neighbourhood diversity (Cardinale et al., 2007; Hong et al., 2021).

**FIGURE 1:**
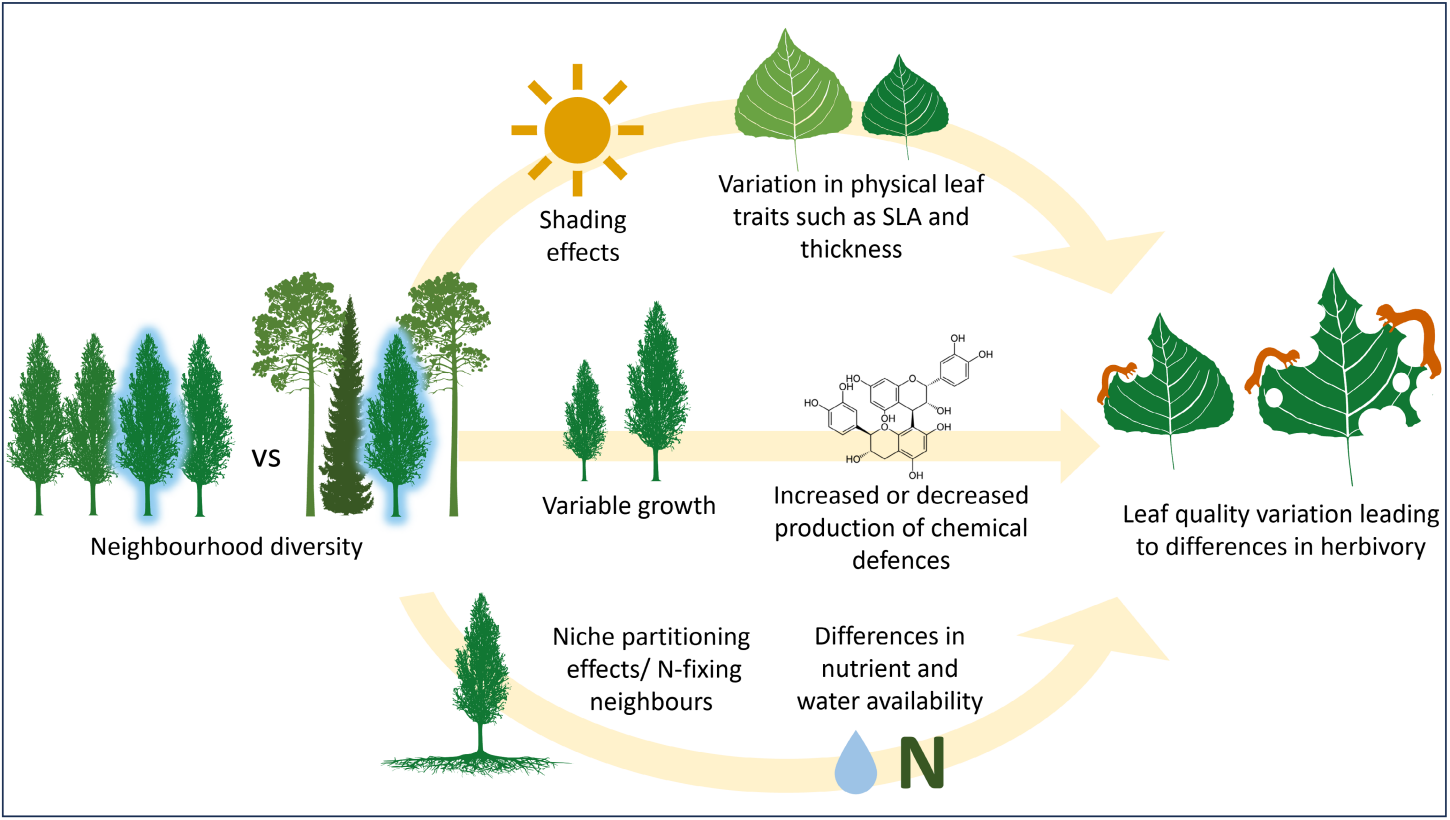
Conceptual diagram showing the ways that neighbourhood diversity can influence leaf quality. Light intensity, nutrient and water availability, and individual tree growth may all vary with neighbourhood diversity, which can cause variation in leaf traits and lead to increased or decreased leaf quality. Differences in leaf quality can in turn lead to variation in herbivory.

Leaf traits are highly plastic to the variation in biotic and abiotic conditions in different plant neighbourhoods due to the distinct morphologies, canopy structures and resource requirements of different species (Callaway et al., 2003; Pretzsch, 2014; Rozendaal et al., 2006). Changes in leaf traits can affect leaf quality to herbivores, and hence increase or decrease the amount of herbivore damage received (Figure 1; Awmack & Leather, 2002; Carmona et al., 2011; Castagneyrol et al., 2018; Moreira et al., 2016; Rosado‐Sánchez et al., 2018b). For example, fast‐growing neighbours in species mixtures can increase canopy stratification and the amount of shading experienced by a focal plant, which might result in a higher specific leaf area (SLA) and lower leaf thickness as an adaptation to maximise photosynthesis in a light‐limited environment (Reich et al., 1997; Roberts & Paul, 2006; Williams et al., 2020). This in turn may increase the palatability of leaves to herbivores, as leaves with higher SLA are more tender and easier to digest (Muiruri et al., 2019). Likewise, the nutritional value of leaves may vary with the availability of nitrogen in the soil, that can be boosted through the presence of neighbouring nitrogen‐fixing plants (N‐fixers) in species mixtures (Richards et al., 2010).

Diverse neighbourhoods may also increase resource‐use complementarity, leading to niche‐partitioning effects, that can reduce competition for space and nutrients. If these mechanisms result in increased resource uptake in species mixtures as compared to monocultures, plants in species mixtures might experience more vigorous growth and increase their investment into chemical and physical defences (Cardinale et al., 2007; Isbell et al., 2017; Loreau & Hector, 2001; Potvin & Gotelli, 2008). Alternatively, growth‐defence trade‐offs could lead to lower levels of defences in plants with increased vigour (Herms & Mattson, 1992); however, evidence for such trade‐offs in diverse neighbourhoods has been limited (Abdala‐Roberts et al., 2014; Moreira et al., 2014).

Leaf trait variation in response to neighbourhood diversity has increasingly been investigated in grassland and forest diversity experiments, but results have been highly variable, with leaf traits including SLA, phenolic compounds and foliar nitrogen increasing, decreasing, or not changing significantly between focal plants growing in monocultures and species mixtures (Castagneyrol et al., 2019; Kostenko et al., 2017; Poeydebat et al., 2020; Wäschke et al., 2015; Williams et al., 2020). Furthermore, plant ontogeny, planting density and the presence of specific neighbour plants such as N‐fixers can also influence leaf traits and may obscure overall neighbourhood diversity effects (Barton & Koricheva, 2010; Benavides et al., 2019; Guyot et al., 2016; Moreira et al., 2017; Richards et al., 2010; Tobner et al., 2014).

Neighbourhood effects may also depend on the species richness and the phylogenetic diversity of the plant mixture. As species richness increases, so does the number of unique plant–plant interactions and of biotic and abiotic environments experienced by a focal plant. The phylogenetic diversity of a neighbourhood can have similar influences, where more phylogenetically diverse species mixtures (e.g. pine‐oak mixture, as opposed to a mixture of two oak species) are predicted to harbour more heterogeneous biotic and abiotic environments due to the greater diversity of plant niches and growth patterns (Jactel et al., 2021; Williams et al., 2020).

To identify general patterns of trait responses to neighbourhood diversity, we conducted a meta‐analysis of studies that compared leaf traits in monocultures and species mixtures. We assessed the responses of leaf thickness, toughness, leaf dry matter content (LDMC), terpenoids, phenolics, carbon (C), SLA and nitrogen (N). We chose to focus on traits that have been shown to influence chewing insects as they have received the most attention in neighbourhood diversity studies (Jactel et al., 2021). We expect that increases of SLA and N would increase leaf quality for chewers, whereas increases of the other six traits assessed would decrease leaf quality (Farmer, 2014; Gardarin et al., 2014; Schädler et al., 2003).

Sources of variation in leaf trait responses were elucidated by assessing the influences of plant species richness, phylogenetic diversity, presence of nitrogen‐fixers, planting density, ontogeny and experimental design in meta‐regression models. Our analysis aimed to answer the following questions:

- Does leaf trait expression differ for plants growing in species mixtures compared to those growing in monocultures?
- Does the direction and/or magnitude of response to neighbourhood diversity differ between individual leaf traits?
- Do leaf trait responses to neighbourhood diversity depend on plant density, species richness, phylogenetic diversity, presence of nitrogen‐fixing neighbours, ontogeny and experimental design?

## 2 MATERIALS AND METHODS

### 2.1 Literature search and screening

SCOPUS and the Web of Science Core Collection were searched for relevant publications in January 2021 using the following search string:

(“plant” OR “tree” OR “crop”) AND (“divers*” OR “intercrop*” OR “species rich*” OR “monoculture” OR “polyculture” OR “cultivar mixture*” OR “neighbo?r*”) AND (“VOC” OR “defen?e” OR “trichome” OR “secondary metabolite*” OR “leaf chemi*” OR “plant quality*” OR “phytochem*” OR “volatile*” OR “resistance” OR “leaf trait” OR “plant trait”) AND “herbivor*”.

Articles published in English were retained, yielding 2381 and 2064 results from the two databases, respectively. A further 24 papers were identified through checking the reference lists of papers identified through the database search, as well as from relevant review papers. Moreover, the list of publications on the TreeDivNet Website (https://treedivnet.ugent.be/index.html) was checked, and members of the network were sent requests for unpublished data. This yielded 18 additional papers and data sets. Finally, several studies included in a previous meta‐analysis by Richards et al. (2010) that had investigated foliar nitrogen levels of trees in monocultures and species mixtures were integrated into this meta‐analysis.

All article titles and abstracts were screened, and irrelevant studies where leaf traits were not measured were excluded. The full text of the remaining articles was then examined, and studies that fitted the following inclusion criteria were retained to be used in the meta‐analysis (see Figure S1 in Supporting Information).

- Plant traits that could influence herbivory were measured on undamaged leaves for a focal plant species growing within monocultures and species mixtures, with other factors such as plant ontogeny, time of year and stand density remaining constant between different plots. Only studies on constitutive leaf traits were considered.
- Mean values of trait measurements, standard errors or standard deviations and sample sizes were reported in the paper or in the supplementary information or were available upon request from the authors.
- Data were gathered from a minimum of two replicate plots for monocultures and each species mixture.

While the original literature search extended to all plant traits, the majority of relevant papers provided data on leaf traits and hence the subsequent analysis was restricted to plant diversity effects on eight leaf traits: SLA, leaf dry matter content (LDMC), thickness, toughness, total nitrogen (N), total carbon (C), phenolic compounds and terpenoid compounds. The canopy layer from which leaves were sampled differed between studies (e.g. lower branches, sun leaves or a mixture of different positions) but was consistent between monoculture and species mixture sampling within each study. Phenolics and terpenoids represent large classes of plant secondary compounds that share a common biosynthetic pathway; in our analysis terpenoids include data on monoterpenes, sesquiterpenes, diterpenes and iridoid glycosides, whereas phenolics include flavonoids, lignins, condensed tannins, hydrolysable tannins and measurements of total phenolics. Due to insufficient data, responses of individual compounds could not be considered; however, there were sufficient effect sizes to examine the effects of neighbourhood diversity on the four subgroups of phenolic compounds mentioned above as well as ‘total phenolics’.

To investigate sources of variation among effect sizes, data for the following moderators were also extracted from each publication: plant species richness for each species mixture; planting density (only for woody plants); study design (experimental vs observational); plant ontogenetic stage (only for woody plants); and presence of nitrogen‐fixing species in a mixture. Additionally, the identity of all focal and neighbouring species within each study was used to calculate average phylogenetic diversity values for each plot (see Methods S1 for details).

### 2.2 Effect size calculations

All statistical analyses were conducted in R version 4.04 (R Core Team, 2021) using the package *metafor* version 3.4 (Viechtbauer, 2010). Effect sizes were calculated as a standardised mean difference (SMD, Hedges' *g*; Gurevitch & Hedges, 1993) between the mean value of a leaf trait of a focal species in a species mixture and that in a monoculture. Positive SMD values indicated that the leaf trait value was higher for focal plants growing in species mixtures compared with monocultures. As we expected the direction of the effect to be highly context‐dependent (i.e. different neighbours may cause either an increase or a decrease in the same leaf trait), we also calculated absolute value effect sizes (hereafter referred to as absolute effect sizes) by removing the sign from all SMD values. This allowed us to compare the magnitude of the effect of neighbourhood diversity on different plant traits.

If traits were measured for a focal plant species in several different mixture types (e.g. monoculture, 2, 4 and 8‐species mixtures) then the same monoculture values would be used as a control for each of the mixture types. When data were presented on a graph, mean values and SD/SE were extracted using the software WebPlotDigitizer (https://automeris.io/WebPlotDigitizer/). When only standard errors were reported, they were transformed to standard deviations by multiplying them by the square root of the sample size.

If studies reported correlations between leaf trait values and plant species richness instead of mean values for monocultures and species mixtures, SMD (d) and variance (*V*_d_) values were approximated using the following formulae derived from Borenstein (2009) (Methods S1). A total of 1007 effect sizes from 43 studies were included in the final meta‐analysis. Distribution of directional and absolute effect sizes for each trait was visualised using orchard plots (Nakagawa et al., 2021).

### 2.3 Meta‐analysis

Multilevel model analysis was performed using the ‘rma.mv’ function in *metafor*. Study ID, experimental site, individual effect ID and plant species were included as random factors to control for non‐independence among effect sizes (Table S11; Nakagawa et al., 2017; Noble et al., 2017). To account for phylogenetic nonindependence arising from relatedness among focal species, the R package *rotl* (Michonneau et al., 2016) was used to create a phylogenetic correlation matrix of all focal species in the meta‐analysis that was then linked to an additional phylogeny random factor (Cinar et al., 2022; Nakagawa & Santos, 2012).

The overall effect of neighbourhood diversity on each leaf trait of a focal plant species was assessed by calculating the grand mean effect sizes of the SMD. An effect was considered significant if the 95% confidence intervals did not overlap with zero (Koricheva et al., 2013). To explore sources of heterogeneity, moderators were incorporated into analysis models for traits with sufficient numbers of effect sizes (Nakagawa et al., 2017), which in this study included C, N, SLA, LDMC, and phenolics. Moderator interactions were not included due to insufficient sample sizes.

Absolute effects of neighbourhood diversity on leaf traits were calculated by repeating the meta‐analysis and meta‐regression models with the sign removed from all effect sizes. This technique has been utilised in previous meta‐analyses to compare the magnitudes of effects where the direction of effects was variable (e.g. Bailey et al., 2009; Champagne et al., 2016; Clements et al., 2022) and was used here to assess the degree of plasticity of different leaf traits in response to neighbourhood diversity, regardless of the direction of response.

Publication bias for each trait type was assessed by constructing funnel plots and inspecting them for asymmetry. In addition, we ran meta‐regression models with sampling error or publication year as moderators to test for small study biases and decline effects, respectively (Nakagawa et al., 2022). Potential biases due to over‐represented plant species were investigated by calculating the proportion of effect sizes derived from each plant species; those that contributed >10% of effect sizes for a specific trait were considered to be over‐represented. Sensitivity analyses were then run to test the impact of these species by testing whether the results changed when these species are excluded from analysis.

## 3 RESULTS

### 3.1 Description of the dataset

Phenolics, N, C, LDMC and SLA were the leaf traits most reported in studies looking at the effects of plant species richness (Table 1). Neighbourhood diversity was experimentally manipulated in most studies (85% of the data) and the majority of data (90% of effect sizes from 32 studies) came from studies on trees, with only 10% of effect sizes from 11 studies reporting effects of neighbourhood diversity on leaf traits in herbaceous plants. A total of 125 focal plant species were represented in the dataset, but silver birch (*Betula pendula*) was highly over‐represented and contributed 26% of all effect sizes. Studies exploring effects of plant diversity on leaf traits had an uneven global distribution, with 57% of effect sizes coming from temperate biomes, 16% from boreal biomes and 27% from tropical and subtropical biomes (mainly from the subtropical BEF‐China experiment, see Figure S2 for details).

**TABLE 1:** Mean directional and absolute effect sizes showing standardised mean differences in leaf traits between diverse neighbourhoods and monocultures.

Trait	*k* (*N*)	Effect, 95% CI	95% PI	Absolute effect, 95% CI	Absolute 95% PI
Thickness	20 (3)	−0.05 [−1.69; 1.60]	[−2.85; 2.76]	0.72 [−0.24; 1.68]	[−0.87; 2.31]
Toughness	20 (3)	−0.40 [−0.72; −0.08]*	[−0.72; −0.08]	0.44 [0.12; 0.75]**	[0.12; 0.75]
LDMC	119 (9)	−0.10 [−0.70; 0.51]	[−1.94; 1.74]	0.66 [0.35; 0.98]***	[0.12; 1.21]
SLA	251 (17)	***0.46* [*−0.03***; ***0.95*]**	[−1.53; 2.45]	1.04 [0.72; 1.35]***	[0.16; 1.91]
Terpenoids	24 (6)	−0.12 [−1.06; 0.82]	[−2.01; 1.77]	0.70 [0.15; 1.26]*	[−0.28; 1.68]
Phenolics	228 (13)	−0.07 [−0.27; 0.13]	[−0.72; 0.58]	0.51 [0.36; 0.65]***	[0.20; 0.81]
Nitrogen	206 (27)	***0.23* [*−0.03***; ***0.49*]**	[−1.04; 1.50]	0.83 [0.53; 1.13]***	[0.10; 1.57]
Carbon	139 (11)	−0.08 [−0.34; 0.18]	[−1.07; 0.92]	0.68 [0.53; 0.83]***	[0.53; 0.83]

*Note*: Effects were considered significant if 95% confidence intervals (95% CI) did not overlap with zero. 95% PI—prediction interval that estimates the range in which effect sizes of 95% of future studies would be expected to fall, *N*—number of studies from which data was extracted for each trait data, *k*—number of individual effect sizes for each trait. Significant effects are shown in bold, marginally significant effects in italics. *** significant at the 0.001 level, ** significant at the 0.01 level, * significant at the 0.05 level

### 3.2 Mean directional and absolute effects of neighbourhood diversity on plant traits

**FIGURE 2:**
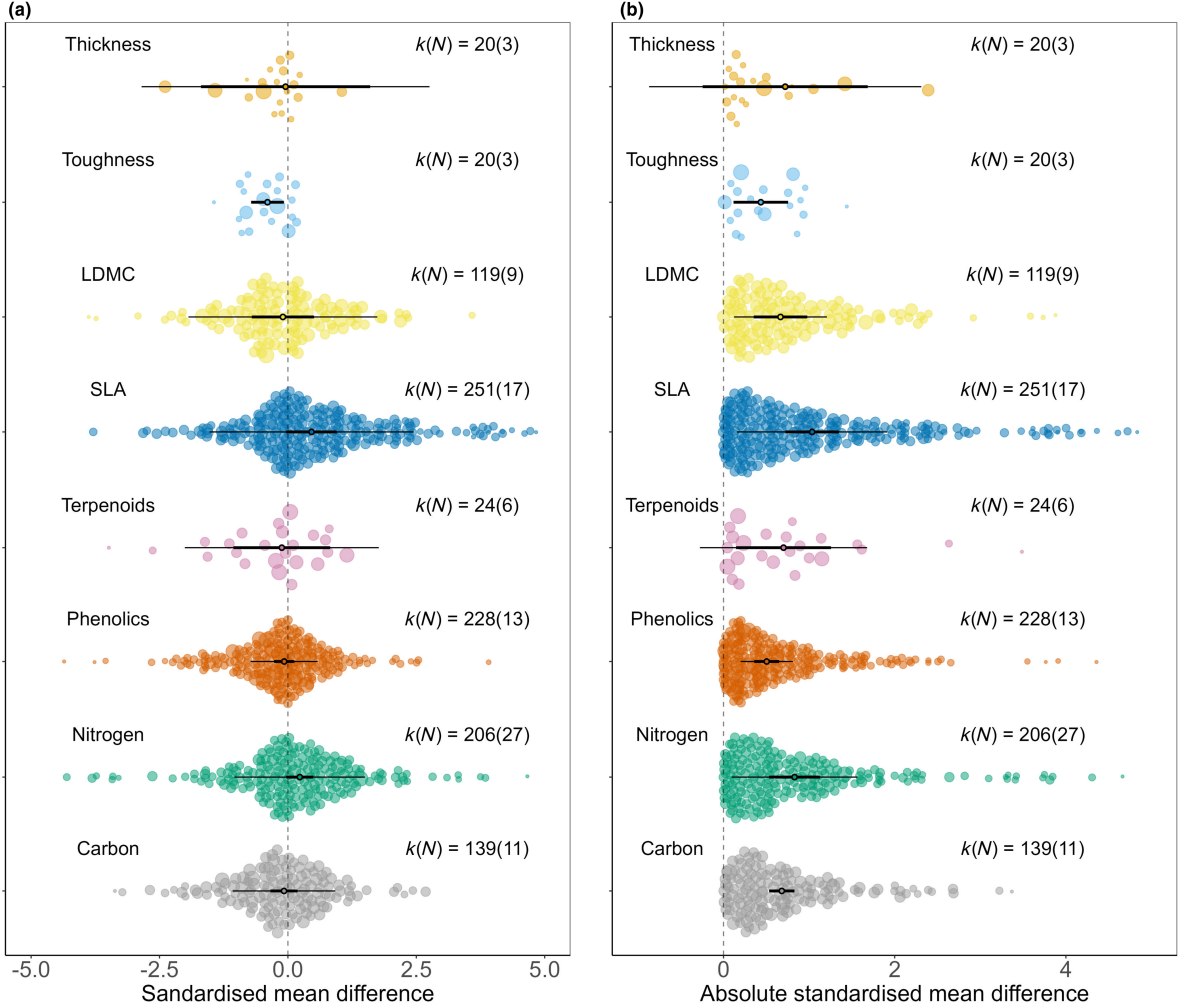
Orchard plots of the directional (a) and absolute (b) effects of neighbourhood diversity on eight leaf traits. *N*—number of studies from which data was extracted for each trait, *k*—number of individual effect sizes for each trait, thick bars—95% confidence intervals (95% CI), thin bars—95% prediction intervals. Effects are considered significant if the 95% CI does not overlap with zero.

Leaf toughness was the only leaf trait that displayed a significant directional change with plant diversity; focal plant leaves were on average tougher in monocultures than in species mixtures, whereas SLA and leaf nitrogen both showed a marginally significant positive response to neighbourhood diversity (Table 1, Figure 1a). The 95% prediction intervals for most traits were broad, showing a high level of heterogeneity. When phenolics were analysed separately by class, none of the phenolic classes showed significant directional responses to neighbourhood diversity, although total phenolics exhibited a marginally significant reduction (Figure 2).

**FIGURE 3:**
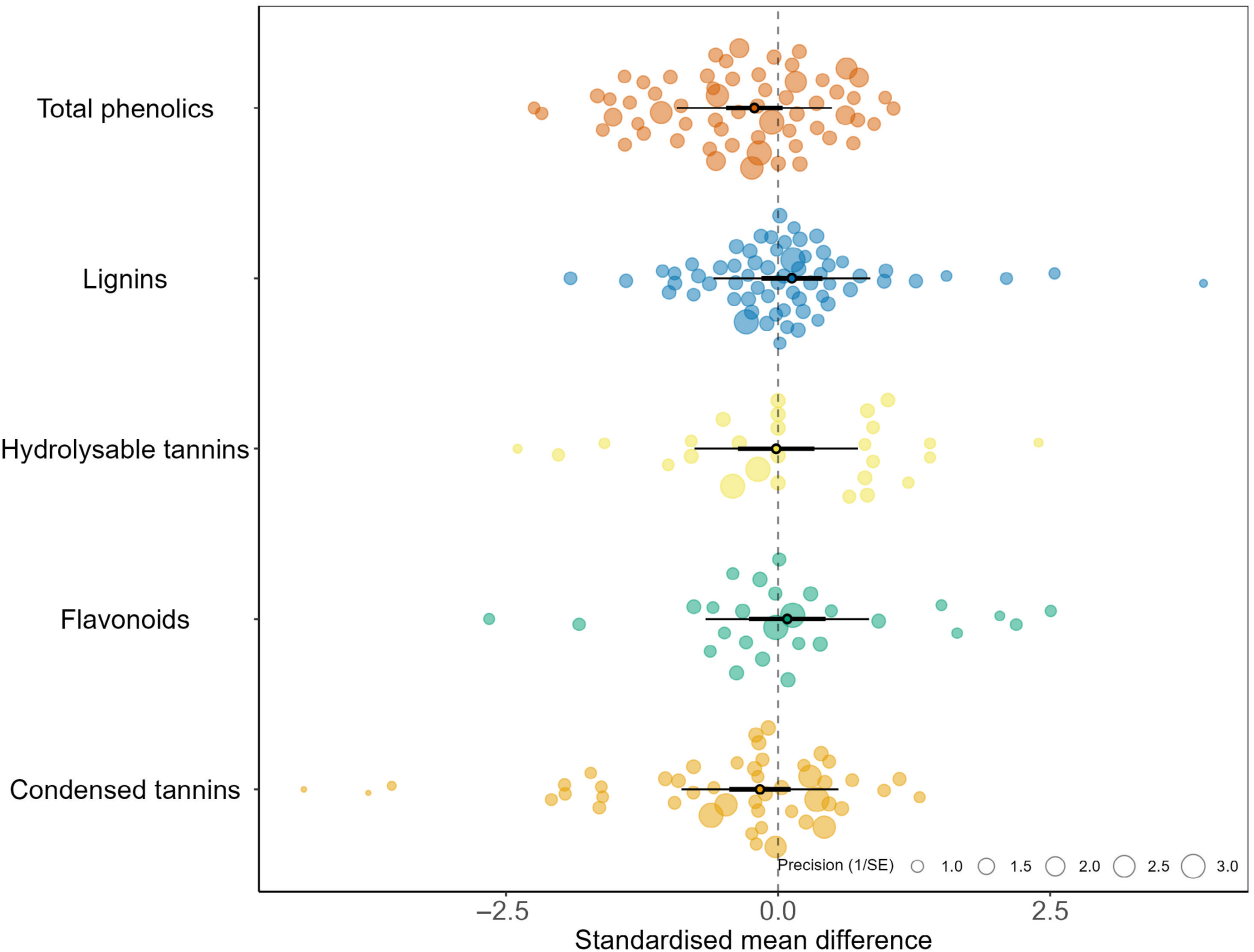
Orchard plots of the directional effects of neighbourhood diversity on five classes of phenolic compounds. Thick bars—95% confidence intervals (95% CI), thin bars—95% prediction intervals. Effects are considered significant if the 95% CI does not overlap with zero.

Analysis of absolute effect sizes showed that all leaf traits apart from leaf thickness exhibited significant differences between monocultures and mixtures (Table 1, Figure 1b). The largest absolute effects were seen for SLA followed by N, whereas leaf toughness and phenolics showed the smallest absolute changes (Table 1; Figure 3).

### 3.3 Meta‐regressions for directional effects

**FIGURE 4:**
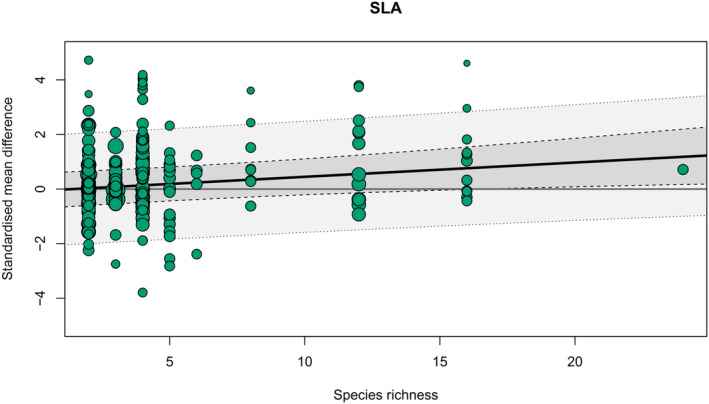
Effect of the species richness of a mixture on the standardised mean difference value for SLA. Black line—slope of the effect, dark grey area—95% confidence interval, light grey area—95 % prediction interval.

The only continuous variable that had a significant effect was species richness, where the positive effects of neighbourhood diversity on SLA were significantly stronger in mixtures with higher species richness (Table S2, Figure 4). SLA also showed significantly different responses depending on study type and tree age and was increased in diverse neighbourhoods in both experimental studies (Table S5) and studies of juvenile trees (Table S4). Nitrogen was likewise increased in mixtures of juvenile trees but, contrary to SLA, was significantly higher in mixed stands only in observational studies—although this is likely a statistical artefact due to low sample sizes. Focal trees in neighbourhoods containing N‐fixers had decreased levels of phenolics and increased N levels as compared to monocultures (Table S3, Figure 4).

### 3.4 Meta‐regressions for absolute effects

**FIGURE 5:**
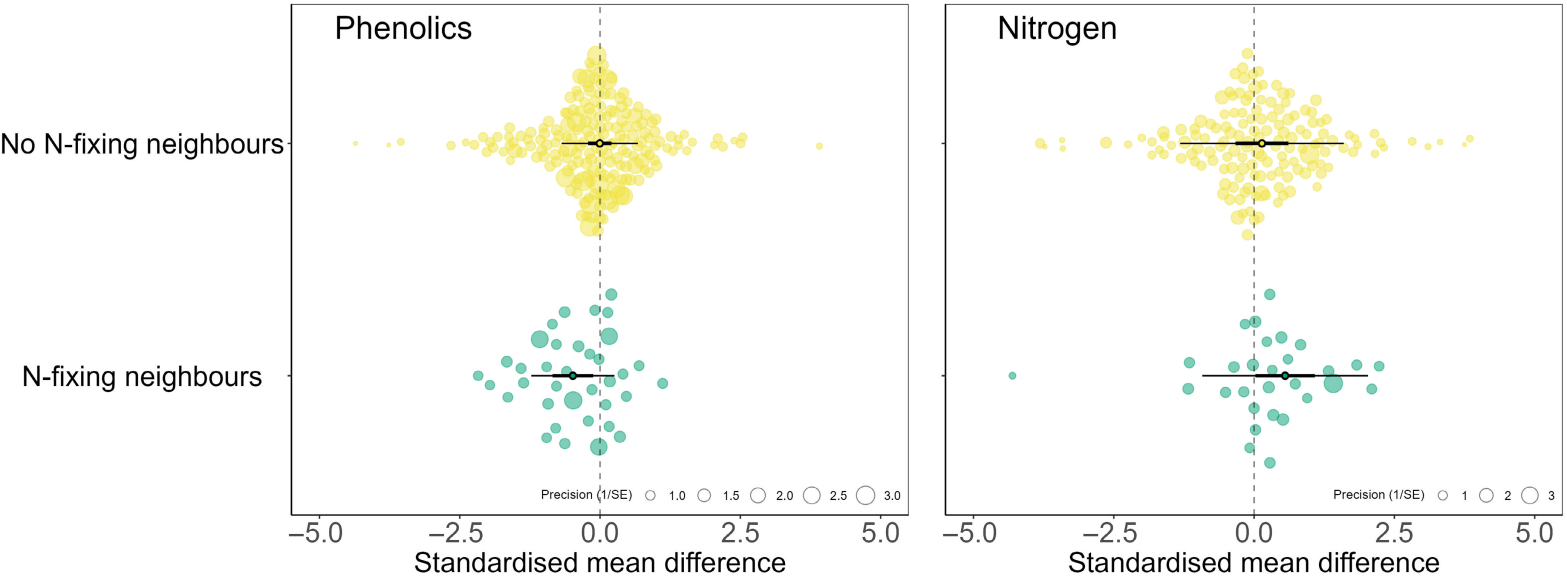
Orchard plots of the effects of neighbourhood diversity on phenolics and leaf nitrogen in the presence and absence of N‐fixing neighbours. Thick bar—95% confidence interval (95% CI), thin bar—95% prediction interval. Effects are considered significant if the 95% CI does not overlap with zero.

Absolute effect sizes for SLA and phenolics were significantly larger in experimental studies than in observational studies (Table S10), and effects on SLA exhibited marginally significant positive relationship with phylogenetic diversity (Table S7). Plant density, species richness, ontogenetic stage and the presence of N‐fixing species had no significant effects on absolute magnitudes of leaf trait responses to neighbourhood diversity (Tables S7–S10; Figure 5).

### 3.5 Sensitivity analysis and publication bias

Due to the uneven distribution of moderators across studies, several of the categorical moderators were confounded. For instance, most studies that included N‐fixing neighbours were experimental, and very few were observational. To account for this, meta‐regressions were repeated with subgroups of effect sizes that were limited to one set of categorical moderators at a time (e.g. by comparing leaf trait responses to neighbourhood diversity in mature and juvenile trees only in experimental forests without N‐fixing neighbours). There were no significant differences between these subsets and the meta‐regressions that used the full data set (results not shown), suggesting that confounded moderators did not lead to any erroneous conclusions.

Studies on silver birch (*Betula pendula*) were over‐represented in this meta‐analysis and contributed >10% of effect sizes for LDMC, C, N, phenolics, toughness and thickness. When *B. pendula* was excluded, the reduction of leaf toughness in species mixtures was no longer significant, but the decrease in phenolics became marginally significant. The mean absolute effect for leaf toughness was also no longer significant when *B. pendula* effect sizes were excluded from analyses, while the mean absolute effect for leaf thickness became significant (see Table S10).

The removal of *B. pendula* effect sizes from meta‐regressions on phenolics changed the outcome of several models; phenolics in mature trees were significantly reduced in species mixtures (Juvenile = −0.04 [−0.23; 0.16] *k* = 34, Mature = −0.23 [−0.44; −0.02], *k* = 33, Qm = 1.812, *p* = 0.178), while increasing species richness had a marginal negative effect (intercept = −0.13 [−0.27; 0.01], *k* = 75). Phenolics remained significantly reduced in the presence of N‐fixing species when *B. pendula* effect sizes were excluded (N‐fixing = −0.38 [−0.70; −0.06] *k* = 12, no N‐fixing = −0.08 [−0.22; 0.06] *k* = 63, Qm = 2.818, *p* = 0.093). Moreover, the difference in response of absolute effects for phenolics to neighbourhood diversity in observational and experimental studies was no longer significant when *B. pendula* effect sizes were removed (*Q*_m_ = 2.677, *p* = 0.102). Meta‐regression results for LDMC, C and N were not affected by the removal *B. pendula* effect sizes.

Visual inspection of funnel plots revealed no major asymmetries (Figure S3); however, the relationship between effect sizes and sampling error was significantly negative for phenolics and significantly positive for N and SLA (Figure S4a). No significant changes in effect sizes with publication year were detected for any trait (Figure S4b).

## 4 DISCUSSION

All but one of the examined leaf traits showed significant absolute differences between monocultures and species mixtures, indicating high phenotypic plasticities of both physical and chemical leaf traits in response to neighbourhood diversity. However, the only trait that displayed a significant mean directional response to neighbourhood diversity was leaf toughness. Taken together, these results suggest that the magnitude and direction of leaf trait responses to plant diversity are highly context‐dependent and may contribute to either increased or decreased leaf quality for herbivores depending on the identity of the focal and neighbouring species.

### 4.1 Individual leaf trait responses to neighbourhood diversity

The largest absolute effects were observed for SLA and N, followed by C and LDMC, whereas smaller changes occurring for leaf toughness, phenolics and terpenoids. The high plasticity of SLA to neighbourhood diversity may reflect responses to light variation, where decreased light availability typically leads to greater SLA and thus greater light capture per unit mass, and the reverse occurs in high light conditions (Chapin et al., 2011; Reich et al., 1997; Williams et al., 2020). Increased canopy stratification and shading in species mixtures could increase SLA in shorter plants, while fast growing species such as *Betula* spp. may conversely experience higher SLA in monocultures where they are self‐shaded by conspecifics (Poeydebat et al., 2020). Leaf dry matter content, toughness and thickness are also known to vary with light levels (Valladares & Niinemets, 2008), albeit to a lesser extent than SLA (Rozendaal et al., 2006), which may explain their lower absolute mean effect sizes. Furthermore, different light conditions can also mediate variation in carbon‐based chemical defences including phenolics and terpenoids, as well as total carbon, as a function of photosynthesis rates (Koricheva et al., 1998; Roberts & Paul, 2006).

While we found no significant differences between the neighbourhood diversity effects on different classes of phenolic compounds, significant variation in direction of response was observed in each group. To further explore this variation, future studies would benefit from including more detailed analysis of secondary metabolites, ideally making use of techniques that can identify specific compounds as has been done by chemical ecologists working in related fields (e.g. metabolomic‐type approaches used by Sedio et al., 2017; Walker et al., 2022).

### 4.2 Predictors of the leaf trait shifts

Both shading and niche partitioning effects have been found to intensify at higher species richness levels (Davrinche & Haider, 2021; Pretzsch, 2014), which might offer an explanation for the increased response of SLA with species richness. While lower relative plasticities could explain the lack of response from other traits to species richness, effect sizes from plant neighbourhoods with high species richness (>6) were derived from only four studies, thereby limiting the extent to which species richness effects could be examined.

Neighbourhood diversity effects in observational studies were expected to be weaker than in experimental studies due to reduced control of confounding environmental variables and the imperfect composition of monoculture plots (monocultures in observational studies are often defined as stands containing >80%–90% of a given species). This was the case for SLA and phenolics, which showed significantly stronger absolute responses to neighbourhood diversity in experimental studies than they did in observational studies. Moreover, the directional shift in SLA was significantly higher in experimental studies than it was in observational studies.

Plant ontogenetic stage influences the expression of leaf traits and defences in plants (Barton & Koricheva, 2010) and may have an interactive effect with neighbourhood diversity (Moreira et al., 2017). The observed decrease in leaf phenolics in species mixtures of mature but not juvenile trees when over‐represented *B. pendula* effect sizes were excluded suggests that phenolic compounds in mature trees are more responsive to neighbourhood effects. Alternatively, decreased phenolics in mature mixed stands of trees could result from stronger shading and complementarity effects relative to those in juvenile stands (Jucker et al., 2020; Lohbeck et al., 2013); however, this is not supported by the responses of both SLA and N, both of which were significantly increased in species mixtures composed of juvenile trees but not mature trees.

Leaf traits were predicted to be more responsive to neighbourhood diversity in stands of high density due to increased shading effects and tree–tree interactions (Pretzsch, 2014; Tobner et al., 2014). Although no overall effect of density was found in this analysis, much of the high‐density data was taken from studies of juvenile trees that may not have grown large enough for canopy closure and notable niche‐partitioning effects to occur.

Species mixtures with high phylogenetic diversity were also predicted to have a greater influence on leaf traits, as distantly related species are more likely to occupy different ecological niches, which could minimise competition and promote niche‐partitioning effects. No significant effects of phylogenetic diversity on leaf trait responses to neighbourhood diversity were found in our analysis, possibly because the phylogenetic diversity score method used in our models may have missed important functional distinctions between closely related species (e.g. deciduous English oak and evergreen Holm oak). Life‐history strategy (e.g. pioneer vs. late successional species) and shade tolerance have been used in other studies to gain insights into the influence of functional diversity (Niinemets & Valladares, 2006; Rüger et al., 2020; Williams et al., 2020); however, a lack of available data for all the focal species considered in primary studies included in our meta‐analysis prevented the inclusion of these metrics into meta‐regression models.

In agreement with a previous meta‐analysis by Richards et al. (2010), leaf nitrogen was significantly increased in diverse neighbourhoods that contained N‐fixers. Conversely, phenolics were reduced in plants growing in neighbourhoods containing N‐fixers, which could be interpreted as evidence of growth‐defences trade‐offs, although only partial support for interspecific growth‐defence trade‐offs has been found in studies included in this meta‐analysis that also measured plant growth (Moreira et al., 2014; Rosado‐Sánchez et al., 2018b; Walter et al., 2012).

### 4.3 Implications of leaf trait shifts in species mixtures

Our study showed that the response of leaf traits to neighbourhood diversity is highly heterogeneous and may contribute to either increased or decreased leaf quality for herbivores, depending on the context. When paired with meta‐analyses by Barbosa et al. (2009) and Jactel et al. (2021) that found that insect herbivory and abundance is on average lower in species mixtures than in monocultures, our findings indicate that leaf trait variation is not a dominant mechanism in mediating reductions in herbivory between diverse neighbourhoods.

However, despite finding overall negative effects of neighbourhood diversity effects on herbivory, both meta‐analyses by Barbosa et al. (2009) and Jactel et al. (2021) demonstrated high degrees of heterogeneity and revealed numerous instances of increased herbivory and herbivore abundance in species mixtures. Our findings may offer novel insights here, as we revealed several circumstances where trait variation in diverse neighbourhoods could positively influence leaf quality for herbivores. For instance, increased SLA in mixtures with high species richness, or increased N and decreased phenolics in neighbourhoods containing N‐fixers could increase the leaf quality of a focal plant and potentially offset the negative effects of reduced plant apparency and increased predation from natural enemies. The advantages of increased leaf quality could be particularly strong for generalist herbivores, which are often less sensitive to neighbourhood diversity effects due to a broader diet range, and may even benefit from a mixed diet (Jactel et al., 2021).

In addition to resistance to herbivory, leaf trait variation may also contribute to differences in plant fitness and productivity in different neighbourhood types (Davrinche & Haider, 2021; Proß et al., 2021; Zeugin et al., 2010). Plants in diverse neighbourhoods often exhibit increased productivity compared to those in monocultures (Feng et al., 2022; Tilman et al., 2001), which might in part be due to a shift towards more acquisitive leaf trait profiles that maximise photosynthesis and growth (e.g. high SLA and N, low LDMC, C and phenolic defences). We found only partial evidence of an acquisitive trait shift in diverse neighbourhoods, with SLA increasing with species richness and phenolics decreasing and N increasing in certain neighbourhood types (e.g. with N‐fixers). Davrinche and Haider (2021) recently assessed the leaf trait responses of 16 tree species in a subtropical diversity experiment and found that immediate conspecific neighbours shifted leaf traits into an acquisitive direction more strongly than neighbourhood diversity on a plot‐level, which may partially explain why evidence for this phenomenon varied in this meta‐analysis.

### 4.4 Future work

This meta‐analysis was limited to the examination of eight leaf traits as there were insufficient data available on other defensive and nutritional leaf traits such as alkaloids and sugar content (Table S1), as well as on other plant parts. Although seminal biodiversity studies were conducted in grasslands (Tilman et al., 2001), studies addressing effects of neighbourhood diversity on leaf traits of herbaceous plants are under‐represented in the literature, and several of the models in this analysis had to be restricted to data on trees. Finally, the genotypic diversity of a neighbourhood may have similar effects on plant traits to species diversity, but received insufficient attention in the literature to be considered in this study (but see Hoeber et al., 2017; Moreira et al., 2014; Weih et al., 2021).

We encourage future studies to explore the areas highlighted above, and to further investigate diverse neighbourhoods with characteristics that were under‐represented in our meta‐regression models, (mature trees, high species richness levels, high phylogenetic diversity).

More broadly, a deeper understanding of neighbourhood diversity effects on leaf traits could be gained if researchers were to account functional diversity within different species mixtures, such as differences in life‐history strategies and shade tolerance, in addition to including measurements of abiotic factors know to effect leaf traits including light availability and soil moisture.

## AUTHOR CONTRIBUTIONS

Julia Koricheva and Juri A. Felix designed this research; Juri A. Felix extracted data from studies and conducted the analysis; Juri A. Felix and Julia Koricheva wrote the manuscript with contributions from Philip C. Stevenson.

## CONFLICT OF INTEREST STATEMENT

Julia Koricheva is an associate editor of Functional Ecology, but took no part in the peer review and decision‐making processes for this paper.

## Supporting information


**Figure S1.** PRISMA diagram showing the identification of relevant articles for this meta‐analysis.
**Figure S2.** Map of study locations.
**Figure S3.** Funnel plots for the eight measured leaf traits.
**Figure S4.** Regressions of directional effect size against sampling error and publication year for LDMC, SLA, phenolics, nitrogen and carbon.
**Methods S1.** Additional information on the moderators which were used in meta‐regression models and the equations used to estimate SMD and variance from data sources in the form of regressions.
**Table S1.** List of all leaf traits extracted from studies and their potential impacts on herbivory.
**Table S2.** Meta‐regression results for directional effects with continuous moderators.
**Table S3.** Meta‐regression results for directional effects in mixtures with and without N‐fixing neighbours.
**Table S4.** Meta‐regression results for the effects of tree ontogeny on directional effect sizes.
**Table S5.** Meta‐regression results for the effects of type of study on directional effect sizes.
**Table S6.** Meta‐regression results for absolute effects with continuous moderators.
**Table S7.** Meta‐regression results for absolute effects in mixtures with and without N‐fixing neighbours.
**Table S8.** Meta‐regression results for the effects of tree ontogeny on absolute effect sizes.
**Table S9.** Meta‐regression results for the effects of study type on absolute effect sizes.
**Table S10.** Sensitivity analysis excluding *Betula pendula* results.
**Table S11.** Measures of components of variance *σ* caused by random factors in meta‐analysis models.

## Data Availability

The data and code used in this meta‐analysis are available on Zenodo (https://zenodo.org/record/8349185).
