# Supplementary material for: Plant neighbourhood diversity effects on leaf traits: A meta‐analysis
Source: Funct Ecol. 2023 Sep 29;37(12):3150–63. doi: 10.1111/1365-2435.14441 (PMC10946959; doi:10.1111/1365-2435.14441)
Supplement: Supplementary file 1 — Figure S1. PRISMA diagram showing the identification of relevant articles for this meta‐analysis. Figure S2. Map of study locations. Figure S3. Funnel plots for the eight measured leaf traits. Figure S4. Regressions of directional effect size against sampling error and publication year for LDMC, SLA, phenolics, nitrogen and carbon. Methods S1. Additional information on the moderators which were used in meta‐regression models and the equations used to estimate SMD and variance from data sources in the form of regressions. Table S1. List of all leaf traits extracted from studies and their potential impacts on herbivory. Table S2. Meta‐regression results for directional effects with continuous moderators. Table S3. Meta‐regression results for directional effects in mixtures with and without N‐fixing neighbours. Table S4. Meta‐regression results for the effects of tree ontogeny on directional effect sizes. Table S5. Meta‐regression results for the effects of type of study on directional effect sizes. Table S6. Meta‐regression results for absolute effects with continuous moderators. Table S7. Meta‐regression results for absolute effects in mixtures with and without N‐fixing neighbours. Table S8. Meta‐regression results for the effects of tree ontogeny on absolute effect sizes. Table S9. Meta‐regression results for the effects of study type on absolute effect sizes. Table S10. Sensitivity analysis excluding Betula pendula results. Table S11. Measures of components of variance σ caused by random factors in meta‐analysis models. [file FEC-37-3150-s001.docx]

Plant Neighbourhood Diversity Effects on Leaf Traits: A Meta-analysis - Supporting information

**Juri A. Felix^1,2^, Philip C. Stevenson^2^ and Julia Koricheva^1^**

^1^ Department of Biological Sciences, Royal Holloway University of London, Egham, UK ^2^Royal Botanic Gardens, Kew, UK.

**Fig. S1** PRISMA diagram showing the identification of relevant articles for this meta-analysis. **Table S1** List of all leaf traits extracted from studies and their potential impacts on herbivory.

**Methods S1** Additional information on the moderators which were used in meta-regression models and the equations used to estimate SMD and variance from data sources in the form of regressions.

**Fig. S2** Map of study locations.

**Table S2.** Meta-regression results for directional effects with continuous moderators.

**Table S3.** Meta-regression results for directional effects in mixtures with and without N-fixing neighbours.

**Table S4.** Meta-regression results for the effects of tree ontogeny on directional effect sizes.

**Table S5.** Meta-regression results for the effects of type of study on directional effect sizes.

**Table S6.** Meta-regression results for absolute effects with continuous moderators.

**Table S7.** Meta-regression results for absolute effects in mixtures with and without N-fixing neighbours.

**Table S8.** Meta-regression results for the effects of tree ontogeny on absolute effect sizes.

**Table S9.** Meta-regression results for the effects of study type on absolute effect sizes.

**Table S10.** Sensitivity analysis excluding *Betula pendula* results.

**Table S11:** Measures of components of variance σ caused by random factors in meta-analysis models.

**Fig. S3** Funnel plots for the eight measured leaf traits.

**Fig. S4** Regressions of directional effect size against sampling error and publication year for LDMC, SLA, phenolics, nitrogen and carbon.

**References**

**
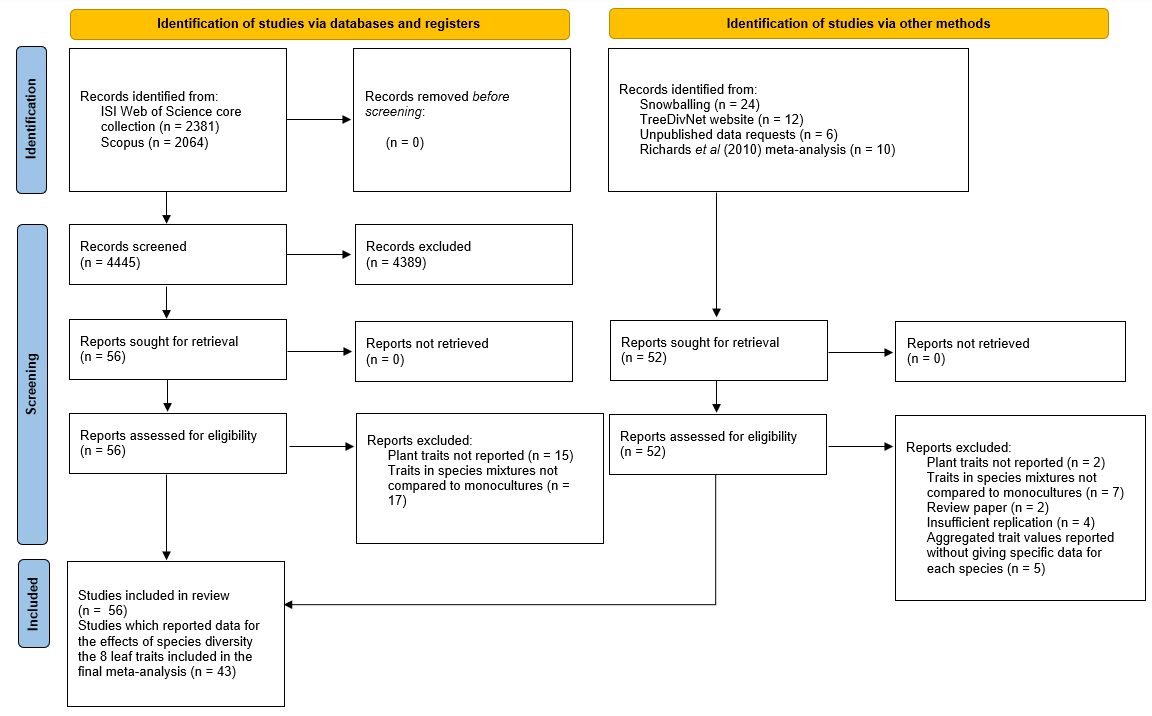
Figure S1:** Preferred Reporting Items for Systematic reviews and Meta-Analyses (PRISMA) diagram showing the identification of relevant articles for this meta-analysis. Diagram template from Page et al, (2021).

| Trait | Impact on herbivory | Reference |
| --- | --- | --- |
| Thickness | Thicker leaves are more resistant to chewing damage. | (Caldwell et al., 2016) |
| Toughness | Tougher leaves are more resistant to chewing damage. | (Malishev & Sanson, 2015; Onoda et al., 2011) |
| LDMC (leaf dry matter content) | Ratio of dry mass to fresh mass. Leaves with higher LDMC are less digestible. | (Gardarin et al., 2014) |
| SLA (specific leaf area) | Ratio of fresh leaf area to dry mass. Leaves with higher SLA tend to be more tender and have lower investment into physical defences. | (Pérez-Harguindeguy et al., 2013; Reich et al., 1997) |
| Trichome density***** | Greater density of trichomes is associated with a reduction of chewing damage. | (Frederickson et al., 2013) |
| Terpenoids | Various actions which increase with concentration: toxic to herbivores, form physical barriers as resins, indirect defence by signalling to parasitoids. | (Farmer, 2014; Mithöfer & Boland, 2012) |
| Alkaloids***** | Toxic to generalist herbivores, more effective at higher concentrations. | (Farmer, 2014; Mithöfer & Boland, 2012) |
| Phenolics | Condensed and hydrolysable tannins act as deterrents and oxidative stressors to insects, flavonoids act as feeding deterrents, and lignins reduce permeability and digestibility of cell walls. | (Barbehenn & Constabel, 2011; DeGabriel et al., 2008) |
| Glucosinolates***** | Form mustard oils upon degradation which deter herbivores. | (Ishida et al., 2014) |
| Nitrogen | Leaves with higher N content are more nutritious and preferred by herbivores. | (Chapin et al., 2011; Minkenberg & Ottenheim, 1990) |
| Carbon | Negatively correlated with Leaf N and palatability to herbivores. | (Schädler et al., 2003) |
| Structural carbohydrates***** | Correlated with leaf toughness and unpalatability. | (Kitajima et al., 2012) |
| Sugars***** | High sugar concentration desirable to herbivores. | (Awmack & Leather, 2002) |
| Protein* | High protein concentration desirable to herbivores. | (Awmack & Leather, 2002) |
| Volatile Organic Compounds (VOCs)* | VOCs can deter insects directly, recruit parasites and parasitoids for top-down defences, or signal to other plant parts/ neighbours to prime other defensive traits. | (Unsicker et al., 2009) |

**Table S1:** List of all leaf traits extracted from studies and their potential impacts on herbivory. Traits which were not included in the final analysis due to a lack of relevant studies are labelled with *.

**Methods S1**

To investigate sources of heterogeneity among effect sizes, information on the following moderators was also derived from each study:

**Species richness:** The total number of different plant species (including the focal plant) growing within each species mixture plot.

**Planting density:** Number of plants per m^2^. A higher planting density was expected to intensify neighbourhood diversity effects, which could in turn lead to greater variation in leaf traits.

**Phylogenetic diversity:** Previous studies have shown that species richness effects differ in mixtures of closely related species compared to mixtures of phylogenetically distant species (Jactel et al., 2021). To account for this, a phylogenetic diversity score (PD) ranging from 0-1 was calculated for each species mixture, where a score of 0 would represent a monoculture (no phylogenetic diversity) and 1 would represent a focal species surrounded by most phylogenetically distant species within the dataset (i.e. in our dataset, an oak tree surrounded by pines).

The identity of all focal and neighbour species in the meta-analysis was used to construct a phylogenetic correlation matrix using the R package ‘rotl’ (Michonneau et al., 2016). This provided phylogenetic correlation scores of 0-1 for all possible species pairs. PD values for each plot were then calculated using the formula: *(Equation S1)*

$$PD =1- \sum(x_{i}c_{i})$$

Where *x_i_* = the fraction of neighbouring species *i* within a mixture, and *c_i_* = correlation score between the focal species and neighbouring species *i*.

**Study type:** Studies were categorised as observational (natural or seminatural stands of plants of varying neighbourhood diversity) or experimental (greenhouse, common garden, or field experiments where plant species richness has been experimentally manipulated). Experimental studies typically have a greater control of abiotic and biotic factors, therefore the effects of neighbourhood diversity could be more apparent than they are in observational studies.

**Ontogenetic stage:** Woody plants were categorised as either juvenile or mature. This information was either given in the article by the authors (i.e., trees described as young or sapling were categorised as juvenile, trees described as adult or reproductive were categorised as mature) or was assigned by judgement of plant/study age at the time of the experiment (e.g. 16 year old silver birch trees were categorised as mature, 5-6 year old white spruce trees were categorised as juvenile). Juvenile trees have access to fewer resources and will encounter different growth limiting factors, so may express different leaf traits than mature trees (Barton & Koricheva, 2010). Herbaceous plants were not considered for this moderator.

**Presence of nitrogen-fixing neighbours:** Whether any of the species present within a mixture was nitrogen-fixing (e.g. in the family Fabaceae, or in the genus *Alnus*). A previous meta-analysis by Richards et al. (2010) showed that leaf nitrogen was significantly increased for trees growing in species mixtures which contained N-fixing neighbours. If the focal species was N-fixing, then a species mixture was not considered to have N-fixing neighbours (unless all additional admixed species were also N-fixing).

The following equations were used to approximate standardised mean difference (d) and variance (V_d_) values from studies which reported correlations between leaf trait values and plant species richness instead of giving mean values for monocultures and species mixtures:

$$d= \frac{2r}{\sqrt{1- r^{2}}}V_{d} and V_{d} = \frac{4V_{r}}{{(1-r^{2})}^{3}} where {V_{r}= \frac{{(1-r^{2})}^{2}}{n-1}}$$

**
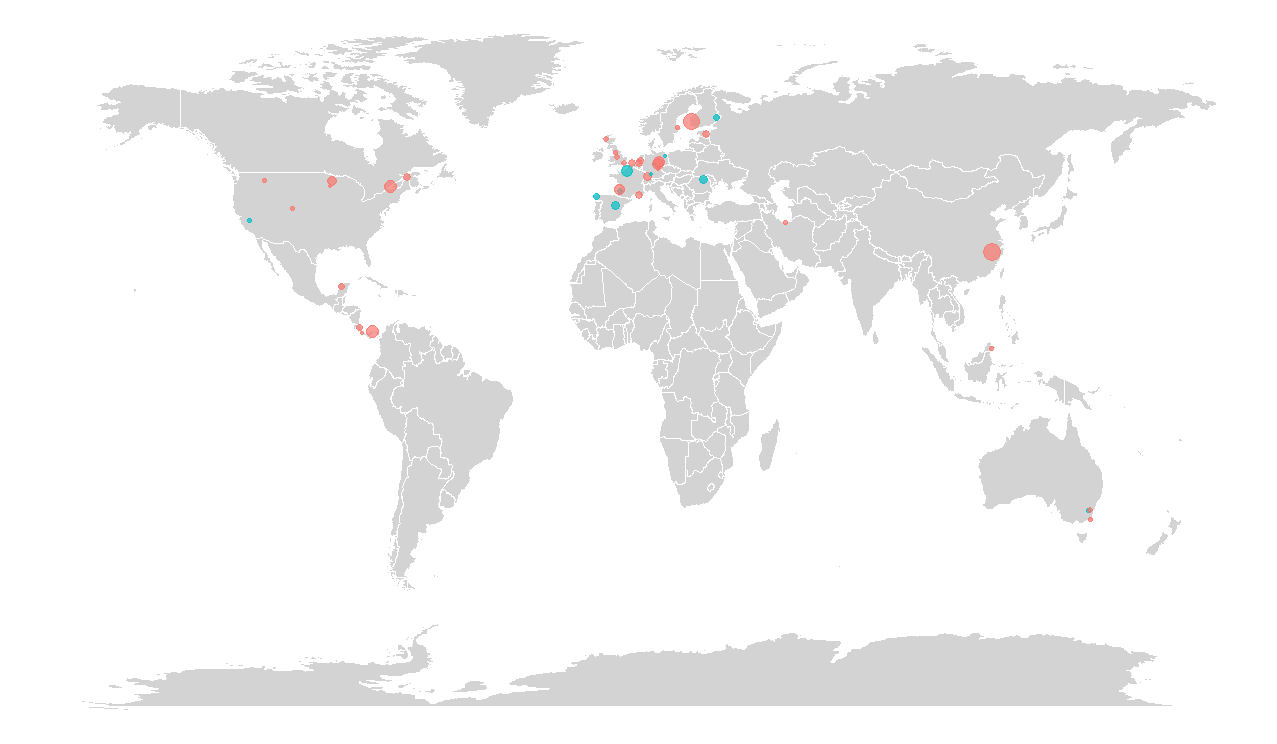
Figure S2:** Map showing locations of the studies in this meta-analysis. Circle size is scaled to the number of effect sizes taken from each experimental site, red circles = experimental studies, blue circle = observational studies.

| Trait | Moderator | Slope , 95 % CI | *k* | Q_m_ | p |
| --- | --- | --- | --- | --- | --- |
| LDMC | Density | -1.76 [−4.05; 0.54] | 79 | 2.255 | 0.133 |
|  | Species richness | -0.03 [−0.08; 0.02] | 119 | 1.184 | 0.277 |
|  | Phylogenetic diversity | -0.32 [−1.47; 0.83] | 118 | 0.302 | 0.583 |
| SLA | Density† | 0.17 [−2.65; 3.40] | 73 | 0.059 | 0.808 |
|  | Species richness | **0.05 [0.01; 0.09]*** | 219 | **6.049** | **0.014** |
|  | Phylogenetic diversity | 0.53 [−0.25; 1.31] | 218 | 1.762 | 0.184 |
| Phenolics | Density | 0.12 [−0.05; 0.28] | 201 | 1.903 | 0.168 |
|  | Species richness | 0.02 [−0.07; 0.11] | 228 | 0.153 | 0.695 |
|  | Phylogenetic diversity | -0.11 [−0.73; 0.50] | 202 | 0.120 | 0.729 |
| Nitrogen | Density† | 0.18 [−0.39; 0.76] | 129 | 0.396 | 0.529 |
|  | Species richness | -0.01 [−0.06; 0.04] | 174 | 0.191 | 0.662 |
|  | Phylogenetic diversity | -0.29 [−1.19; 0.61] | 142 | 0.402 | 0.526 |
| Carbon | Density† | -0.09 [−0.81; 0.62] | 110 | 0.066 | 0.797 |
|  | Species richness | -0.02 [−0.06; 0.03] | 139 | 0.550 | 0.458 |
|  | Phylogenetic diversity | -0.72 [−1.74; 0.30] | 118 | 1.903 | 0.168 |

**Table S2:** Meta-regression results with continuous moderators. 95 % CI = 95 % confidence intervals of slope, *k* = number of effect sizes, Q_m_ = test of moderators, p = significance value of Q_m_. †Data from IDENT experiments was removed from these models due to their extreme density values which were much higher than density seen from other experiments (bold if significant, italics if marginally significant).

**Table S3:** Effects of neighbourhood diversity on leaf traits in mixtures with and without N-fixing neighbours. 95 % CI = 95 % confidence intervals of each category, *k* = number of effect sizes, Q_m_ = test of moderators, p = significance of Q_m_ (bold if significant, italics if marginally significant).

| **Trait** | N-fixing neighbours | | No N-fixing neighbours | | Qm | p |
| --- | --- | --- | --- | --- | --- | --- |
|  | Effect, 95 % CI | K | Effect, 95 % CI | K |  |  |
| LDMC | 0.14 [−0.89; 1.17] | 8 | -0.11 [−0.72; 0.50] | 111 | 0.317 | 0.574 |
| SLA | 0.41 [−0.41; 1.22] | 25 | 0.33 [−0.33; 1.00] | 202 | 0.047 | 0.828 |
| Phenolics | **-0.48 [−0.85; -0.12] **** | 36 | -0.01 [−0.21; 0.20] | 192 | **7.295** | **0.007** |
| Nitrogen | **0.55 [0.024; 1.08] *** | 29 | 0.14 [−0.33; 0.62] | 153 | **5.140** | **0.023** |
| Carbon | 0.04 [−0.78; 0.87] | 8 | -0.08 [−0.35; 0.19] | 131 | 0.314 | 0.575 |

**Table S4:** Effects of neighbourhood diversity on leaf traits in juvenile and mature trees. 95 % CI = 95 % confidence intervals of each category, *k* = number of effect sizes, Q_m_ = test of moderators, p = significance of Q_m_ (bold if significant, italics if marginally significant).

| **Trait** | Juvenile | | Mature | | Qm | p |
| --- | --- | --- | --- | --- | --- | --- |
|  | Effect, 95 % CI | K | Effect, 95 % CI | K |  |  |
| LDMC | -0.45 [−1.29; 0.39] | 53 | 0.14 [−0.58; 0.86] | 66 | 1.237 | 0.266 |
| SLA | **0.55 [0.04; 1.07] *** | 124 | 0.06 [−0.49; 0.61] | 79 | *2.879* | *0.090* |
| Phenolics | -0.01 [−0.24; 0.22] | 135 | *-0.25 [−0.54; 0.04]* | 85 | 2.452 | 0.117 |
| Nitrogen | **0.25 [0.04; 0.46] *** | 123 | 0.26 [−0.14; 0.67] | 43 | **0.005** | **0.947** |
| Carbon | -0.14 [−0.39; 0.11] | 102 | 0.13 [−0.32; 0.57] | 37 | 1.328 | 0.249 |

**Table S5:** Effects of neighbourhood diversity on leaf traits in experimental and observational studies. 95 % CI = 95 % confidence intervals of each category, *k* = number of effect sizes, Q_m_ = test of moderators, p = significance of Q_m_ (bold if significant, italics if marginally significant).

| **Trait** | Observational | | Experimental | | Qm | p |
| --- | --- | --- | --- | --- | --- | --- |
|  | Effect, 95 % CI | K | Effect, 95 % CI | K |  |  |
| LDMC | 0.33 [−0.61; 1.27] | 40 | -0.33 [−1.03; 0.36] | 79 | 1.340 | 0.247 |
| SLA | -0.31 [−1.04; 0.42] | 54 | **0.62 [0.10; 1.13] *** | 197 | **6.954** | **0.008** |
| Phenolics | -0.10 [−0.62; 0.41] | 28 | -0.06 [−0.29; 0.16] | 200 | 0.019 | 0.890 |
| Nitrogen | **0.80 [0.12; 1.48] *** | 15 | *0.17 [−0.03; 0.37]* | 191 | *3.201* | *0.074* |
| Carbon | 0.67 [−0.28; 1.62] | 11 | -0.12 [−0.39; 0.14] | 128 | 2.563 | 0.109 |

**Table S6:** Absolute meta-regression results with continuous moderators. 95 % CI = 95 % confidence intervals of slope, *k* = number of effect sizes, Q_m_ = test of moderators, p = significance value of Q_m_ (bold if significant, italics if marginally significant). †Data from IDENT experiments was removed from these models due to their extreme density values which were much higher than density seen from other experiments.

| Trait | Moderator | Absolute slope , 95 % CI | *k* | Q_m_, | p |
| --- | --- | --- | --- | --- | --- |
| LDMC | Density | 0.14 [−0.91; 1.20] | 79 | 0.070 | 0.792 |
|  | Species richness | 0.00 [−0.04; 0.05] | 119 | 0.003 | 0.960 |
|  | Phylogenetic diversity | 0.24 [−0.73; 1.20] | 118 | 0.236 | 0.627 |
| SLA | Density† | -0.79 [−3.00; 1.43] | 73 | 0.486 | 0.486 |
|  | Species richness | 0.03 [-0.01; 0.06] | 219 | 1.546 | 0.214 |
|  | Phylogenetic diversity | *0.56 [−0.06; 1.19]* | 218 | *3.175* | *0.076* |
| Phenolics | Density | 0.05 [-0.05; 0.15] | 201 | 1.161 | 0.281 |
|  | Species richness | 0.04 [-0.05; 0.12] | 228 | 0.761 | 0.383 |
|  | Phylogenetic diversity | 0.08 [−0.48; 0.63] | 202 | 0.070 | 0.791 |
| Nitrogen | Density† | 0.07 [−0.56; 0.69] | 129 | 0.042 | 0.837 |
|  | Species richness | 0.02 [−0.03; 0.06] | 174 | 0.502 | 0.479 |
|  | Phylogenetic diversity | -0.25 [-1.05; 0.56] | 142 | 0.363 | 0.547 |
| Carbon | Density† | 0.07 [−0.41; 0.55] | 110 | 0.080 | 0.777 |
|  | Species richness | 0.00 [−0.04; 0.04] | 139 | 0.001 | 0.982 |
|  | Phylogenetic diversity | 0.50 [−0.33; 1.33] | 118 | 1.395 | 0.238 |

**Table S7:** Absolute effects of neighbourhood diversity on leaf traits in mixtures with and without N-fixing neighbours. 95 % CI = 95 % confidence intervals of each category, *k* = number of effect sizes, Q_m_ = test of moderators, p = significance of Q_m_ (bold if significant, italics if marginally significant).

| **Trait** | N-fixing neighbours | | No N-fixing neighbours | | Qm | p |
| --- | --- | --- | --- | --- | --- | --- |
|  | Absolute effect, 95 % CI | K | Absolute effect, 95 % CI | K |  |  |
| LDMC | *0.75 [-0.01; 1.51]* | 8 | **0.68 [0.36; 0.99]***** | 111 | 0.036 | 0.850 |
| SLA | **1.20 [0.69; 1.71]***** | 25 | **0.91 [0.58; 1.24]***** | 202 | 1.266 | 0.261 |
| Phenolics | **0.55 [0.28; 0.82]***** | 36 | **0.49 [0.34; 0.65]***** | 192 | 0.148 | 0.701 |
| Nitrogen | **0.87 [0.47; 1.28]***** | 29 | **0.80 [0.46; 1.14]***** | 153 | 0.181 | 0.672 |
| Carbon | **0.87 [0.20; 1.53]*** | 8 | **0.67 [0.52; 0.83]***** | 131 | 0.314 | 0.575 |

**Table S8:** Absolute effects of neighbourhood diversity on leaf traits in juvenile and mature trees. 95 % CI = 95 % confidence intervals of each category, *k* = number of effect sizes, Q_m_ = test of moderators, p = significance of Q_m_ (bold if significant, italics if marginally significant).

| **Trait** | Juvenile | | Mature | | Qm | p |
| --- | --- | --- | --- | --- | --- | --- |
|  | Absolute effect, 95 % CI | K | Absolute effect, 95 % CI | K |  |  |
| LDMC | **0.72 [0.31; 1.12]***** | 53 | **0.70 [0.35; 1.05]***** | 66 | 0.009 | 0.923 |
| SLA | **1.05 [0.64; 1.45]***** | 124 | **0.65 [0.27; 1.04]***** | 79 | 2.840 | 0.092 |
| Phenolics | **0.54 [0.35; 0.73]***** | 135 | **0.43 [0.22; 0.64]***** | 85 | 1.010 | 0.315 |
| Nitrogen | **0.70 [0.46; 0.95]***** | 123 | **0.67 [0.30; 1.05]***** | 43 | 0.021 | 0.885 |
| Carbon | **0.67 [0.50; 0.84]***** | 102 | **0.72 [0.43; 1.01]***** | 37 | 0.088 | 0.767 |

**Table S9:** Absolute effects of neighbourhood diversity on leaf traits in experimental and observational studies. 95 % CI = 95 % confidence intervals of each category, *k* = number of effect sizes, Q_m_ = test of moderators, p = significance of Q_m_ (bold if significant, italics if marginally significant).

| **Trait** | Observational | | Experimental | | Qm | p |
| --- | --- | --- | --- | --- | --- | --- |
|  | Absolute effect, 95 % CI | K | Absolute effect, 95 % CI | K |  |  |
| LDMC | **0.80 [0.36; 1.24]***** | 40 | **0.67 [0.30; 1.04]***** | 79 | 0.303 | 0.582 |
| SLA | **0.45 [0.11; 0.80]*** | 54 | **1.13 [0.88; 1.38]***** | 197 | **16.973** | **< 0.001** |
| Phenolics | **0.31 [0.11; 0.51]**** | 28 | **0.60 [0.47; 0.73]***** | 200 | **5.937** | **0.015** |
| Nitrogen | **1.15 [0.63; 1.67]***** | 15 | **0.78 [0.46; 1.11]***** | 191 | 2.057 | 0.152 |
| Carbon | **0.56 [0.06; 1.05]*** | 11 | **0.69 [0.54; 0.85]***** | 128 | 0.263 | 0.608 |

**Table S10:** Results of sensitivity analyses for the effects of *Betula pendula* over-representation. Effect and 95% confidence interval of neighbourhood diversity on the expression of leaf carbon, nitrogen, LDMC, phenolics, thickness and toughness when including all effect sizes, and when excluding *B. pendula*. *k* = number of effect sizes, N = number of studies.

| Trait | Overall effect | | *k* (N) | *B. pendula* excluded effect | | *k* (N) |
| --- | --- | --- | --- | --- | --- | --- |
|  | directional | absolute |  | directional | absolute |  |
| Carbon | -0.08 [-0.34; 0.18] | **0.68 [ 0.53; 0.83] ***** | 139 (11) | -0.03 [−0.49; 0.44] | **0.66 [ 0.50; 0.82]***** | 118 (9) |
| Nitrogen | ***0.23 [-0.03; 0.49]*** | **0.83 [ 0.53; 1.13] ***** | 206 (27) | ***0.23 [-0.03; 0.50]*** | **0.84 [ 0.55; 1.14] ***** | 185 (26) |
| LDMC | -0.10 [−0.70; 0.51] | **0.66 [ 0.35; 0.98] ***** | 119 (9) | 0.00 [−0.76; 0.77] | **0.79 [ 0.44; 1.15] ***** | 98 (7) |
| Phenolics | -0.07 [-0.27; 0.13] | **0.51 [ 0.36; 0.65] ***** | 228 (13) | ***-0.12 [−0.25; 0.00]*** | **0.43 [ 0.28; 0.59] ***** | 75 (10) |
| Thickness | -0.05 [−1.69; 1.60] | 0.72 [−0.24; 1.68] | 20 (3) | -0.33 [−2.67; 2.02] | **1.29 [0.47; 2.10] **** | 4 (2) |
| Toughness | **-0.40 [−0.72; -0.08] *** | **0.44 [ 0.12; 0.75] **** | 20 (3) | -0.34 [−0.81; 0.12] | 0.35 [-0.12; 0.82] | 4 (2) |

**Table S11:** Measures of components of variance σ caused by random factors in meta-analysis models. Random factors responsible for the variance components are as follows: σ experiment = experimental site, σ study = ID of study, σ species = species ID, σ phylo = species ID linked to phylogenetic correlation matrix, σ EF = individual effect size. H2 = estimate of proportion of variation due to phylogenetic random factor and is equivalent to Pagel’s λ (Nakagawa & Santos, 2012).

| **Trait** | **σ experiment** | **σ study** | **σ species** | **σ phylo** | **σ EF** | **H2** |
| --- | --- | --- | --- | --- | --- | --- |
| All | 0.0771 | 0 | 0.1467 | 0.4035 | 0.02789 | 0.62 |
| Thickness | 0 | 0.1879 | 0.1879 | 0.9635 | 0 | 0.72 |
| Toughness | 0 | 0 | 0 | < 0.0001 | 0 | 1.00 |
| Terpenoids | 0 | 0.175 | 0.0001 | 0.4074 | 0.115 | 0.58 |
| LDMC | 0.0001 | 0.561 | 0.2231 | 0 | 0 | 0.00 |
| SLA | 0.0707 | 0.2284 | 0.4115 | 0.071 | 0.1832 | 0.07 |
| C | 0.0202 | 0 | 0.2207 | 0.0004 | 0 | 0.00 |
| N | 0 | 0 | 0.3797 | 0.0221 | 0 | 0.06 |
| phenolics | 0.1006 | 0 | 0 | 0 | 0 | 0.00 |


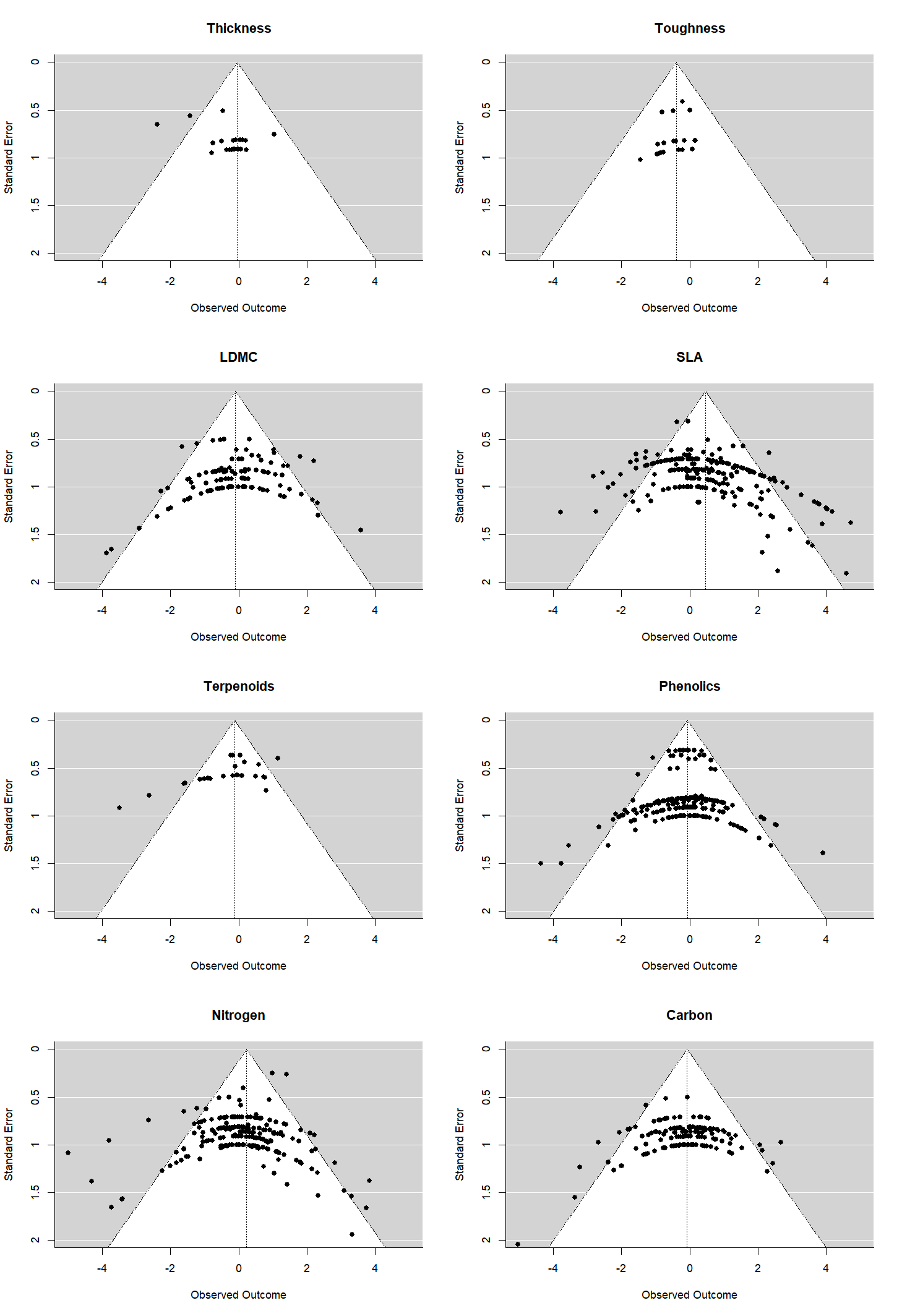
**Figure S3:** Funnel plots for the meta-analysis models of the eight leaf traits. Individual effect sizes values are represented by the x-axis and standard errors by the y-axis, with the white funnel representing the mean value ± 1.96 standard error. Asymmetrical distributions of points may indicate heterogeneity and/or publication bias.

**
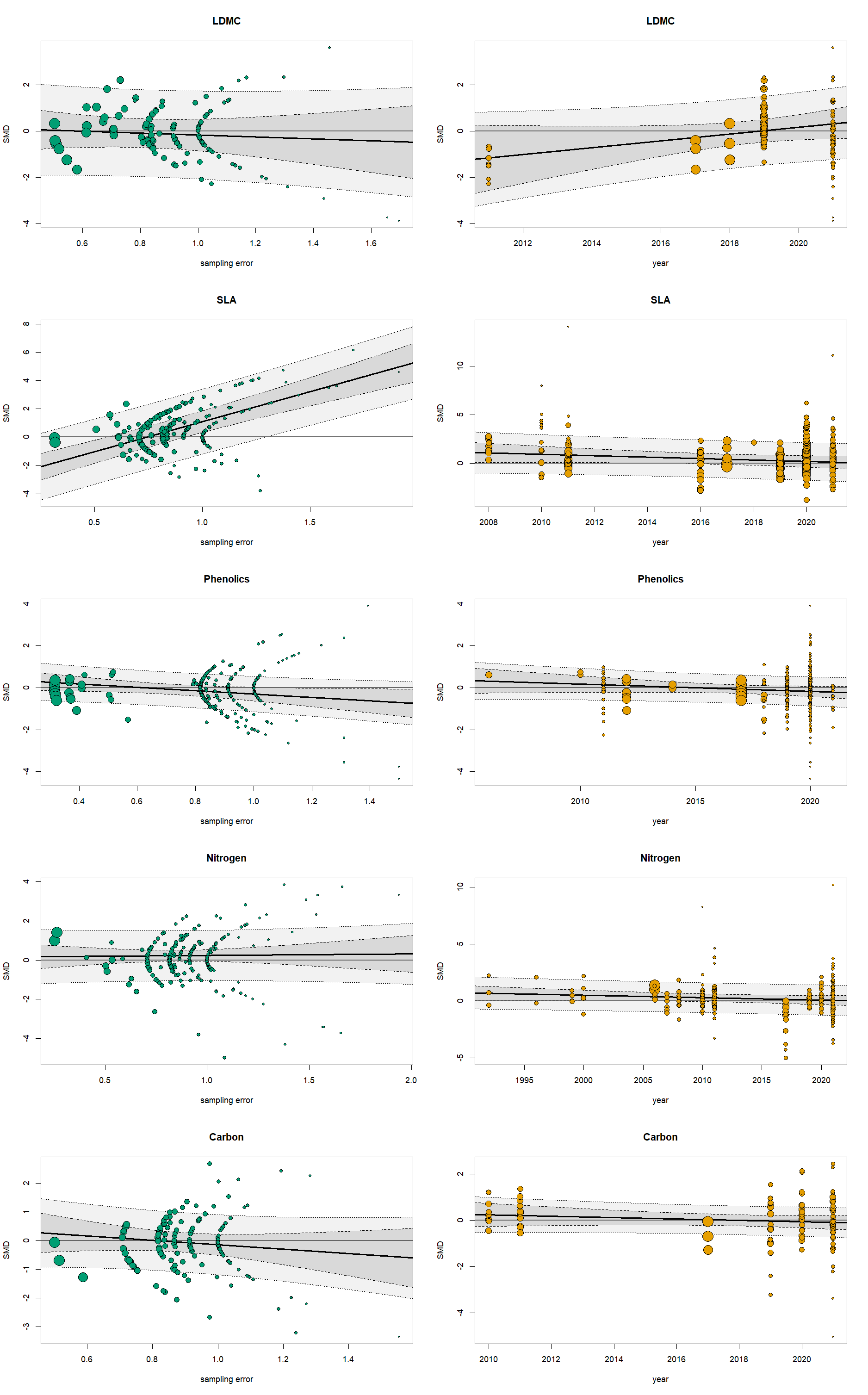
**

**Figure S4:** Meta-regression plots showing the relationship between directional effect sizes and (a) sampling error and (b) publication year for neighbourhood diversity effects on LDMC, SLA, phenolics, nitrogen and carbon. Departure of the SMD from the 95% CI, shown in dark grey, indicates a bias in the effect associated with sampling error or study year, respectively.

**References**

Awmack, C. S., & Leather, S. R. (2002). Host plant quality and fecundity in herbivorous insects. *Annual Review of Entomology*, *47*, 817–844. https://doi.org/10.1146/annurev.ento.47.091201.145300

Barbehenn, R. V., & Peter Constabel, C. (2011). Tannins in plant–herbivore interactions. *Phytochemistry*, *72*(13), 1551–1565. https://doi.org/10.1016/j.phytochem.2011.01.040

Barton, K. E., & Koricheva, J. (2010). The Ontogeny of Plant Defense and Herbivory: Characterizing General Patterns Using Meta‐Analysis. *The American Naturalist*, *175*(4), 481–493. https://doi.org/10.1086/650722

Caldwell, E., Read, J., & Sanson, G. D. (2016). Which leaf mechanical traits correlate with insect herbivory among feeding guilds? *Annals of Botany*, *117*(2), 349–361. https://doi.org/10.1093/aob/mcv178

Chapin, F. S., Matson, P. A., & Vitousek, P. M. (2011). *Principles of terrestrial ecosystem ecology* (2nd ed). Springer.

DeGabriel, J. L., Wallis, I. R., Moore, B. D., & Foley, W. J. (2008). A simple, integrative assay to quantify nutritional quality of browses for herbivores. *Oecologia*, *156*(1), 107–116. https://doi.org/10.1007/s00442-008-0960-y

Farmer, E. E. (2014). *Leaf defence*. Oxford University Press.

Frederickson, M. E., Ravenscraft, A., Hernández, L. M. A., Booth, G., Astudillo, V., & Miller, G. A. (2013). What happens when ants fail at plant defence? Cordia nodosa dynamically adjusts its investment in both direct and indirect resistance traits in response to herbivore damage. *Journal of Ecology*, *101*(2), 400–409. https://doi.org/10.1111/1365-2745.12034

Gardarin, A., Garnier, É., Carrère, P., Cruz, P., Andueza, D., Bonis, A., Colace, M.-P., Dumont, B., Duru, M., Farruggia, A., Gaucherand, S., Grigulis, K., Kernéïs, É., Lavorel, S., Louault, F., Loucougaray, G., Mesléard, F., Yavercovski, N., & Kazakou, E. (2014). Plant trait–digestibility relationships across management and climate gradients in permanent grasslands. *Journal of Applied Ecology*, *51*(5), 1207–1217. https://doi.org/10.1111/1365-2664.12293

Ishida, M., Hara, M., Fukino, N., Kakizaki, T., & Morimitsu, Y. (2014). Glucosinolate metabolism, functionality and breeding for the improvement of Brassicaceae vegetables. *Breeding Science*, *64*(1), 48–59. https://doi.org/10.1270/jsbbs.64.48

Jactel, H., Moreira, X., & Castagneyrol, B. (2021). Tree Diversity and Forest Resistance to Insect Pests: Patterns, Mechanisms and Prospects. *Annual Review of Entomology*, *66*(1), 277–296. https://doi.org/10.1146/annurev-ento-041720-075234

Kitajima, K., Llorens, A.-M., Stefanescu, C., Timchenko, M. V., Lucas, P. W., & Wright, S. J. (2012). How cellulose-based leaf toughness and lamina density contribute to long leaf lifespans of shade-tolerant species. *The New Phytologist*, *195*(3), 640–652. https://doi.org/10.1111/j.1469-8137.2012.04203.x

Malishev, M., & Sanson, G. D. (2015). Leaf mechanics and herbivory defence: How tough tissue along the leaf body deters growing insect herbivores. *Austral Ecology*, *40*(3), 300–308. https://doi.org/10.1111/aec.12214

Michonneau, F., Brown, J. W., & Winter, D. J. (2016). rotl: An R package to interact with the Open Tree of Life data. *Methods in Ecology and Evolution*, *7*(12), 1476–1481. https://doi.org/10.1111/2041-210X.12593

Minkenberg, O. P. J. M., & Ottenheim, J. J. G. W. (1990). Effect of leaf nitrogen content of tomato plants on preference and performance of a leafmining fly. *Oecologia*, *83*(3), 291–298. https://doi.org/10.1007/BF00317551

Mithöfer, A., & Boland, W. (2012). Plant defense against herbivores: Chemical aspects. *Annual Review of Plant Biology*, *63*, 431–450. Scopus. https://doi.org/10.1146/annurev-arplant-042110-103854

Nakagawa, S., & Santos, E. S. A. (2012). Methodological issues and advances in biological meta-analysis. *Evolutionary Ecology*, *26*(5), 1253–1274. https://doi.org/10.1007/s10682-012-9555-5

Onoda, Y., Westoby, M., Adler, P. B., Choong, A. M. F., Clissold, F. J., Cornelissen, J. H. C., Díaz, S., Dominy, N. J., Elgart, A., Enrico, L., Fine, P. V. A., Howard, J. J., Jalili, A., Kitajima, K., Kurokawa, H., McArthur, C., Lucas, P. W., Markesteijn, L., Pérez-Harguindeguy, N., … Yamashita, N. (2011). Global patterns of leaf mechanical properties. *Ecology Letters*, *14*(3), 301–312. https://doi.org/10.1111/j.1461-0248.2010.01582.x

Page, M. J., Moher, D., Bossuyt, P. M., Boutron, I., Hoffmann, T. C., Mulrow, C. D., Shamseer, L., Tetzlaff, J. M., Akl, E. A., Brennan, S. E., Chou, R., Glanville, J., Grimshaw, J. M., Hróbjartsson, A., Lalu, M. M., Li, T., Loder, E. W., Mayo-Wilson, E., McDonald, S., … McKenzie, J. E. (2021). PRISMA 2020 explanation and elaboration: Updated guidance and exemplars for reporting systematic reviews. *BMJ*, *372*, n160. https://doi.org/10.1136/bmj.n160

Pérez-Harguindeguy, N., Díaz, S., Garnier, E., Lavorel, S., Poorter, H., Jaureguiberry, P., Bret-Harte, M. S., Cornwell, W. K., Craine, J. M., Gurvich, D. E., Urcelay, C., Veneklaas, E. J., Reich, P. B., Poorter, L., Wright, I. J., Ray, P., Enrico, L., Pausas, J. G., de Vos, A. C., … Cornelissen, J. H. C. (2013). New handbook for standardised measurement of plant functional traits worldwide. *Australian Journal of Botany*, *61*(3), 167. https://doi.org/10.1071/BT12225

Reich, P. B., Walters, M. B., & Ellsworth, D. S. (1997). From tropics to tundra: Global convergence in plant functioning. *Proceedings of the National Academy of Sciences*, *94*(25), 13730–13734. https://doi.org/10.1073/pnas.94.25.13730

Richards, A. E., Forrester, D. I., Bauhus, J., & Scherer-Lorenzen, M. (2010). The influence of mixed tree plantations on the nutrition of individual species: A review. *Tree Physiology*, *30*(9), 1192–1208. https://doi.org/10.1093/treephys/tpq035

Schädler, M., Jung, G., Auge, H., & Brandl, R. (2003). Palatability, Decomposition and Insect Herbivory: Patterns in a Successional Old-Field Plant Community. *Oikos*, *103*(1), 121–132. https://doi.org/10.1034/j.1600-0706.2003.12659.x

Unsicker, S. B., Kunert, G., & Gershenzon, J. (2009). Protective perfumes: The role of vegetative volatiles in plant defense against herbivores. *Current Opinion in Plant Biology*, *12*(4), 479–485. https://doi.org/10.1016/j.pbi.2009.04.001
